# AKT1^E17K^ Is Oncogenic in Mouse Lung and Cooperates with Chemical Carcinogens in Inducing Lung Cancer

**DOI:** 10.1371/journal.pone.0147334

**Published:** 2016-02-09

**Authors:** Donatella Malanga, Stefania Belmonte, Fabiana Colelli, Marzia Scarfò, Carmela De Marco, Duarte Mendes Oliveira, Teresa Mirante, Caterina Camastra, Monica Gagliardi, Antonia Rizzuto, Chiara Mignogna, Orlando Paciello, Serenella Papparella, Henrik Fagman, Giuseppe Viglietto

**Affiliations:** 1 Dipartimento di Medicina Sperimentale e Clinica, Università Magna Graecia, Catanzaro, Italy; 2 BIOGEM-Istituto di Ricerche Genetiche, Ariano Irpino (AV), Italy; 3 Dipartimento di Scienze della Salute, Unità di Anatomia Patologica, Università Magna Graecia, Catanzaro, Italy; 4 IBFM-CNR, c/o Università Magna Graecia, Catanzaro, Italy; 5 Dipartimento di Scienze Mediche e Chirurgiche, Università Magna Graecia, Catanzaro, Italy; 6 Department of Veterinary Medicine and Animal Productions, Università Federico II, Napoli, Italy; 7 Department of Clinical Pathology and Genetics, Sahlgrenska University Hospital, Göteborg, Sweden; Centro Nacional de Investigaciones Oncológicas (CNIO), SPAIN

## Abstract

The hotspot AKT1^E17K^ mutation in the pleckstrin homology domain of AKT1 occurs in approximately 0.6–2% of human lung cancers. Recently, we have demonstrated that AKT1^E17K^ transforms immortalized human bronchial cells. Here by use of a transgenic Cre-inducible murine strain in the wild type Rosa26 (R26) locus (*R26-AKT1*^*E17K*^ mice) we demonstrate that AKT1^E17K^ is a *bona-fide* oncogene and plays a role in the development of lung cancer *in vivo*. In fact, we report that mutant AKT1^E17K^ induces bronchial and/or bronchiolar hyperplastic lesions in murine lung epithelium, which progress to frank carcinoma at very low frequency, and accelerates tumor formation induced by chemical carcinogens. In conclusion, AKT1^E17K^ induces hyperplasia of mouse lung epithelium *in vivo* and cooperates with urethane to induce the fully malignant phenotype.

## Introduction

Lung cancer is a leading cause of cancer-related deaths, being associated with a 5-year worldwide survival rate of less than 15% [1,2]. Mutational activation of the epidermal growth factor receptor (EGFR) pathway is the major pathogenic event in non tobacco-induced adenocarcinoma (ADC), whereas the pathway driven by v-Ki-ras2/Kirsten rat sarcoma viral oncogene homolog (KRAS) is involved in tobacco-mediated lung carcinogenesis [3–6]. Unfortunately, activating mutations in EGFR are typically not present in lung squamous cell carcinoma (SCC), the second most common type of NSCLC [7], and thus targeted therapy is ineffective. Recent studies showing 2–4% rate of p110 α-catalytic subunit of PI3K (PIK3CA) mutations [8–10] and 1% rate of AKT1 mutations [11,12] in SCC have suggested that targeting these genes may prove a successful therapeutic option [7,13–15].

The AKT kinases (AKT1, AKT2, AKT3) represent the primary downstream end-point of the phosphoinositide 3-kinase (PI3K) pathway, regulating proliferation, survival, metabolism and invasion. AKT1 and AKT2 are frequently activated in human cancer [16–18] following loss of the lipid phosphatase PTEN, activating mutations and/or copy number variation in EGFR or HER2 tyrosine kinase receptors, activating mutations in KRAS, in PIK3CA [19,20]^,^ [21] or in AKT1 itself [22]. In lung cancer a somatic mutation in the pleckstrin homology (PH) domain of AKT1 that results in glutamic acid to lysine substitution at residue 17 (E17K) was reported with overall frequency of 0.6–2% [7,11,12,23–25].

AKT1^E17K^ shows increased affinity for PI(4,5)P2, enhanced plasma membrane recruitment and constitutive activation [22,26]. Endogenous AKT1^E17K^ mutant protein detected in lung cancer cells shows enhanced membrane localization [11], which results in the activation of downstream signalling [11,22].

The role of mutant AKT1^E17K^ in epithelial tumorigenesis remains unclear because studies in cellular models in breast and lung cells have produced discordant results [27–30]. In addition, no murine strain that models the AKT1^E17K^ mutation has been generated so far.

These considerations prompted us to address the role of AKT1^E17K^ in the transformation of lung epithelial cells *in vivo*. Herein, we demonstrate that AKT1^E17K^ promotes hyperplasia in the mouse lung and cooperates with other genetic lesions to induce the full malignancy.

## Materials and Methods

### Vector Design and Generation of transgenic mice

**Fig 1:**
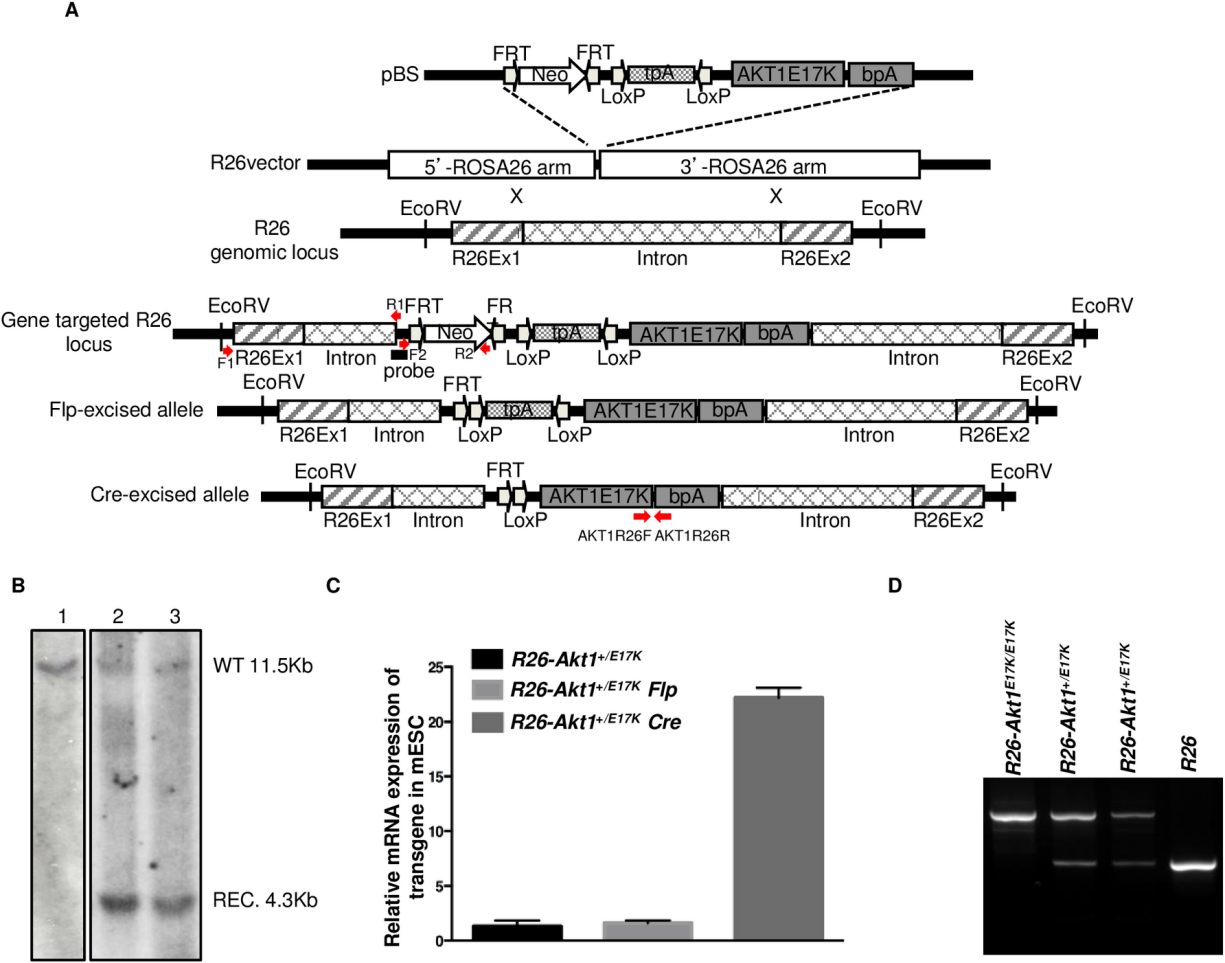
Generation of transgenic *R26-AKT1*^*E17K*^ mouse. **A.** Schematic representation of the targeting construct used for the conditional knock-in in the *R26* locus. The human *AKT1*^*E17K*^ cDNA preceded by a loxP-flanked transcriptional stop cassette, was recombined into the R26 locus. Cre-mediated removal of the stop cassette links Rosa26 exon 1 to the exogenous cDNA allowing expression of the transgene. **B.** Southern blot of EcoRV digested genomic DNA derived from mESCs transfected with the targeting construct carrying mutant AKT1. Lane 1: DNA from non-targeted mESCs, lane 2 and 3: DNA from DNA from two different *R26-AKT1*^*E17K*^ mESCs strains. Endogenous allele corresponds to the 11.5 kb band (*WT*); mutant allele corresponds to the 4.3 kb band (*REC*). **C.** Relative mRNA expression of human AKT1^E17K^ by Q-RT-PCR in targeted mESCs and in the corresponding cells transfected with the Flp (pFlpE-IRES-Puro) or Cre recombinase (pCre-IRES-Puro). Data are from replicate experiments as the mean±SD. ***p<0.001. **D**. Genotype analysis by PCR on tail-tip DNA of genetically modified mice as indicated.

To generate *R26-AKT1*^*E17K*^ transgenic mice human AKT1 cDNA was amplified by PCR from human blood mononuclear cells by PCR using primers 5’-ATGAGCGACGTGGCTATT-3’ and 5’-TCAGGCCGTGCCGCTGGC-3’. The PCR product was cloned into a shuttle plasmid (pBlueScript) using standard cloning techniques. Mutant AKT1^E17K^ cDNA was generated by Quick Change Site-Direct Mutagenesis (Stratagene) and sub-cloned into the pROSA26 vector [31–33] to obtain the targeting construct pR26-AKT1^E17K^, which consisted of 5' and 3' homology arms of the ROSA26 locus, FRT/FRT-flanked neomycin resistance (Neo) cassette and loxP/loxP-flanked triple polyadenylation (tpA) sequence. See Fig 1 for details.

30μg of the R26-AKT1^E17K^ plasmid was linearized with SfiI restriction enzyme (New England, Biolabs) and electroporated into mouse embryonic stem cells (mESC). G418-resistant mESC clones were isolated and screened for correct targeting of the locus by PCR (primers F1 and R1, respectively: 5'-AGGGAACGCAGGGAGACTGAGGTGACCCTTCTTT-3', 5'-GATTGTCTGTTGTGCCCAGTCATAGCCGAATAGC-3) amplifying a 3.9 kb fragment. PCR positive G418-resistant clones were subsequently screened by Southern blot probing for the 5’ recombination junction. Once identified, targeted mESC clones were transfected with Flp-expressing plasmid (pFlpE-IRES-puro) for the excision of the Neo cassette. Subsequently, Neo-deleted mESC clones were isolated, screened by PCR (primers F2 and R2, respectively: 5'-CGGCCTCGACTCTACGATACCGTCGATCC-3', R2 5'-GGATCGAGATCTGATAACTTCGTA-3') and injected into B6 blastocysts, which were subsequently transferred into pseudo-pregnant females to produce chimeric animals at the Embryonic Stem Cell Facility of IRGS (Ariano Irpino, Avellino, Italy). The generated chimeras were bred to establish germline transmission of the human allele. Genotypes of *R26-AKT1*^*E17K*^ mice were determined by PCR using genomic DNA isolated from the tail tips with the following primers: 5'ARMF, 5'-AACTGCAGACTTGTGGGATAC-3'; 3'ARMR, 5'-ATATTAGTCCACCTCACTCCT-3'; mE17KR5 5'-GCCAACCCTCCTTCACAATA-3'. The *R26-AKT1*^*E17K*^ line was mated with *Ttf1-Cre* line (a gift of Dr. Mario De Felice, IRGS, Ariano Irpino, Italy), to generate *R26-AKT1*^*E17K*^*; Ttf1-Cre;* mice. The *Ttf1*-Cre mice were genotyped by using the following primers: FP, 5'-CCTGATGGACATGTTCAGGGACA-3': RP, 5'-GCCAGATCTCCTGTGCAGCATGT-3'. *R26-AKT1*^*E17K*^ mice were back-crossed on B6 background. In all experiments littermates were used as controls.

Animal experimentation was approved by the local ethical committee “Comitato Etico per la Sperimentazione Animale” (CESA) of IRSG and conform to regulations and guidelines of Italy and the European Union. All efforts were made to minimize animal suffering (S1 ARRIVE Checklist). Mice were housed in a highly controlled microbiological environment to guarantee SPF conditions. They were maintained in IVC cages, under constant conditions of temperature (22±2°C), humidity (55%±10 UR) and light/dark cycle of 12/12 hours. The animals had free access to irradiated standard diet and water. Mice were genotyped by PCR of tail DNA [34]. The deletion of loxP-flanked transcriptional stop sequences was determined by PCR using the following primers:

TRF, 5'-GGATCGACGGTATCGTAGAGTCGAGGCCG-3';

L2R 5'-GCCAATGAAGGTGCCATCATTCTTGAGGAGGAAG-3'.

Expression of AKT1^E17K^ in mESCs transfected with pCre-IRES-puro was determined by quantitative RT-PCR.

### Southern blot

A standard protocol for Southern blot was used. Genomic DNA (30 μg) was digested with EcoRV. A 500bp probe on the 5’ side of the targeted insertion site (from CTTGAAAGTGGAGTAACTAC to TCAGAAGCTTTGAACTAGAA in the ROSA26 locus) was labelled with DIG-dUTP, using PCR DIG Probe Synthesis Kit (Roche Diagnostics AG, Rotkreuz, Switzerland). This probe identifies a 11.5 kb fragment in wild type ROSA26 locus and a 4.3kb fragment in targeted ROSA26 locus.

### Western Blot and antibodies

Whole tissue protein extracts were prepared with NP-40 buffer (10 mM Tris–HCl pH 7.5, 150 mM NaCl, 1% NP-40) containing protease inhibitors (SigmaFast, Sigma-Aldrich). Western blot analysis was carried out by standard methods[11]. Anti-phospho-AKT (Ser473) (#4058), anti-AKT1 (#2938), anti-phospho-FoxO1 (Ser256) (#9461), anti-FoxO1 (#2880), anti-phospho-GSK3-α/β (Ser21/9) (#9331), anti-GSK3-α (#9338), anti-GSK3-β (#9332) were purchased from Cell Signaling Technology (Danver, MA).

### Quantitative reverse transcription real-time PCR (Q-RT-PCR)

Total RNA was prepared as described [35]. RT-PCR was performed on RNA extracted by Trizol (Invitrogen) and retro-transcribed with SuperScript II (Invitrogen). Q-RT-PCR was performed using the Power SYBR Green PCR Master Mix in ABI Prism 7900 thermocycler (Applied Biosystems, Foster City, CA). Gene expression was normalised to GAPDH mRNA content. The relative amounts of mRNA or DNA were calculated by the comparative cycle threshold (CT) method [36]. The following primers are used in Q-RT-PCR

AKT1R26 forward 5'-CACACCACCTGACCAAGATG-3';

AKT1R26 reverse 5'-AATCAAGGGTCCCCAAACTC-3'.

### Virus infection of mice

Control *R26 a*nd *R26-AKT1*^*E17K*^ mice were infected with 10^6^ or 10^7^ pfu of Adenoviruses expressing Cre (Ad-Cre) (Vector Biolabs, Philadelphia, PA). Mice between 6 and 12 weeks of age were anesthetized with isoflurane and Ad-Cre was administered intranasally. Mice received 120 μl of Ad-Cre, in two doses of 60 μl each, with a break between the two doses, to allow the mice to recover a normal breathing. Control mice received equal amount of buffer phosphate saline solution (S1 ARRIVE Checklist).

### Experimental Carcinogenesis

*R26 or R26-AKT1*^*E17K*^ littermates were either infected with Ad-Cre (10^6^−10^7^ PFU) or treated with solvent alone before receiving intra-peritoneal injection with a single dose of 1 mg/g of body weight of urethane, an ethyl ester of carbamic acid extensively used in murine models of carcinogenesis [37], dissolved in sterile 0.9% saline solution. Mice were monitored weekly and all efforts were made to minimize suffering. No mouse had to be euthanized prior to the specific experimental end-point (6, 9 and 18 months, respectively) for health reasons. Mice were sacrificed after 6, 9 and 18 months by cervical dislocation (S1 ARRIVE Checklist). Lungs were removed and inflated with 10% neutral buffered formalin. Lung tissues were collected and fixed with 10% formalin, and embedded in paraffin using standard procedures. Lung sectioning was performed to encompass 1.5 mm of the lung parenchyme; frontal sections were collected at 15 levels with 100 μm spacing) of the sagittal distance to adequately cover both peripheral and central regions. Sections were mounted on slides and stained with hematoxylin ad eosin and were evaluated by light microscopy by veterinary pathologists and human pathologist (OP, HF and CM). For each tumor the slide with maximum lesion diameter was identified and recorded under the microscope.

### Histological analysis and immunohistochemistry

Lung tissues were collected and fixed with 10% formalin, and embedded in paraffin using standard procedures. Sections (5μm) were mounted on slides and stained with hematoxylin and eosin to be evaluated by light microscopy by veterinary (OP and HF) or human (CM) pathologists. A combined score of hyperplastic lesions was calculated by adding a score measuring the number of epithelial layers (Layer score) to a score measuring percentage of hyperplasia present in the whole section (Global hyperplasia score). The Layer score was defined as follows: score 1, presence of 1–2 layer(s), score 2, presence of 2–3 layers, score 3, presence of >4 layers. The Global hyperplasia score was defined as follows: score 1, when hyperplasia was present in 1–20% of total sections analysed (3 serial sections/mouse); score 2, when hyperplasia was fond in 21–60% of total sections analysed (3 serial sections/mouse); score 3, when hyperplasia was found in 61–100% of total sections analysed (3 serial sections/mouse).

Immunostaining with anti-phospho-AKT was performed with standard protocols using the Vectastain Universal Quick Kit and DAB Peroxidase Substrate Kit (Vector Laboratories, Burlingame) according to the manufacturer’s instructions. Antigen retrieval was carried out with microwave treatment in antigen unmasking solution (VectorLaboratory, Burlingame, CA) for 10 min. Rabbit anti-phospho-AKT (Ser473) (1:1,000, #4058) was purchased from Cell Signaling Technology.

Immunostaining for Ki67 was performed with standard protocols using Bond™ Polymer Refine Detection (Leica Biosystem, Buffalo Grove, IL) according to the manufacturer’s instructions. Rabbit anti-Ki67 (1:100, PA5-19462) was purchased from Fisher Scientific (Thermo Fisher Sientific, Pittsburgh, PA). Immunostaining for p21 was performed with standard protocols using EnVision™ Detection Systems Dako, according to the manufacturer’s instructions. Rabbit polyclonal Ab-5; anti-p21/WAF1 antiserum (1:50) was purchased from Oncogene Research Products (Cambridge, MA). Ki67 and p21 positivity was determined by counting positive nuclei in 10 fields at X400 magnification.

### Laser capture microdissection (LCM)

Microdissection was performed using the Laser Microdissector Leica LMD6 & LMD7 (Leica Microsystem, Milan). 5-μm tumor tissue sections onto glass slides were inserted into the Laser Microdissector for dissection of lung tumors. Selected areas were captured with infrared laser pulses onto CapSure Macro LCM Caps.

### Direct *Kras* sequencing

Microdissected samples were lysed by SB LysePrep Kit (Silicon Biosystem, Bologna) according to manufacturer' instructions. Aliquots of lysis solution (2.5 μl) were used as template for DNA amplification with the following primers: mKrasF-TCCTAC AGGAAACAAGTA and mKras R-TTATTTATGGCAAATACA. The amplification program used was: 95°C/5 min; 35x (95°C/30 s, 52°C/30 s 72°C/30 s) for denaturation, annealing, and extension; 72°C/7 min to terminate extension, followed by cooling to 15°C. PCR products were separated on 2% agarose gels and purified by the QIAquick PCR purification kit (Qiagen). Purified PCR products were analyzed on 3500 Genetic Analyzer (Life Technologies, Carlsbad, CA, USA). For all analyses, data were aligned to the consensus sequence obtained from the BLAST GenBank database.

### Immunofluorescence staining

Deparaffinized sections were hydrated in decreasing ethanol gradient solutions and rinsed in wash solution (TBST, 0.05 mol/L Tris Buffered Saline with Tween20). Antigen retrieval was performed with citrate buffer pH 6 for 30 minutes at 98°C followed by washing in phosphate-buffered saline (PBS; pH 7.4). Sections were incubated with antibodies against SP-C (goat polyclonal antibody anti-SP-C, 1:200 diluition, Santa Cruz Biotechnologies, Santa Cruz, CA, USA) or CC-10 (goat polyclonal antibody anti-CC-10, 1:200 diluition, Santa Cruz Biotechnologies, Santa Cruz, CA, USA) for 60 min, followed by incubation with secondary Alexa Fluor 488-conjugated anti-goat IgG antibodies (1:400 dilution; Santa Cruz Biotechnology) for 60 min. The cells were counterstained with DAPI (2 μg/mL; Santa Cruz Biotechnology), mounted using anti-fade mounting medium (DakoEnvision System, Inc, CA, USA) and observed at Fluorescence Microscopy (Leica Microsystems).

### Statistical analysis

Data presented are the means ± SD of *n* independent assays or replicates as indicated in the text. Continuous variables were analyzed by Student’s t-test or ANOVA test, while categorical variables were analysed by χ^2^ or Fisher’s exact tests. Significance was calculated by Log-rank (Mantel-Cox) test (GraphPad Prizm 5 Software, San Diego, CA).

## Results

### Mutant AKT1^E17K^ promotes hyperplasia of bronchi and bronchioli

We generated a transgenic mouse strain (*R26-AKT1*^*E17K*^) that conditionally expresses human mutant AKT1 in the lung by use of a knock-in system (Cre conditional *Rosa26*, *R26*) that harbours loxP-flanked transcriptional stop sequences upstream of mutant AKT1 cDNA (Fig 1A). Murine ESCs were targeted and screened by Southern blot to identify clones that carried the recombinant allele (Fig 1B) and for Cre-induced expression of the transgene (Fig 1C). Cre-responsive *R26-AKT1*^*E17K*^ mESCs were then used to generate chimeric mice. Germline transmission of the knocked-in allele was confirmed by PCR (Fig 1D).

To express mutant AKT1 in lung epithelium we used two different systems: intranasal instillation of an Adenovirus carrying the Cre recombinase (Ad-Cre) or mating of *R26-AKT1*^*E17K*^ mice with *Ttf1-Cre* mice [38,39]. The use of *Ttf1-Cre* mice allowed expression of the transgene in the bronchial epithelium [39]^,^ [40] whereas instillation of Ad-Cre presents the advantages of allowing the somatic activation of the transgene in patched areas and of not selecting in advance the cell type in which the transgene is expressed.

**Fig 2:**
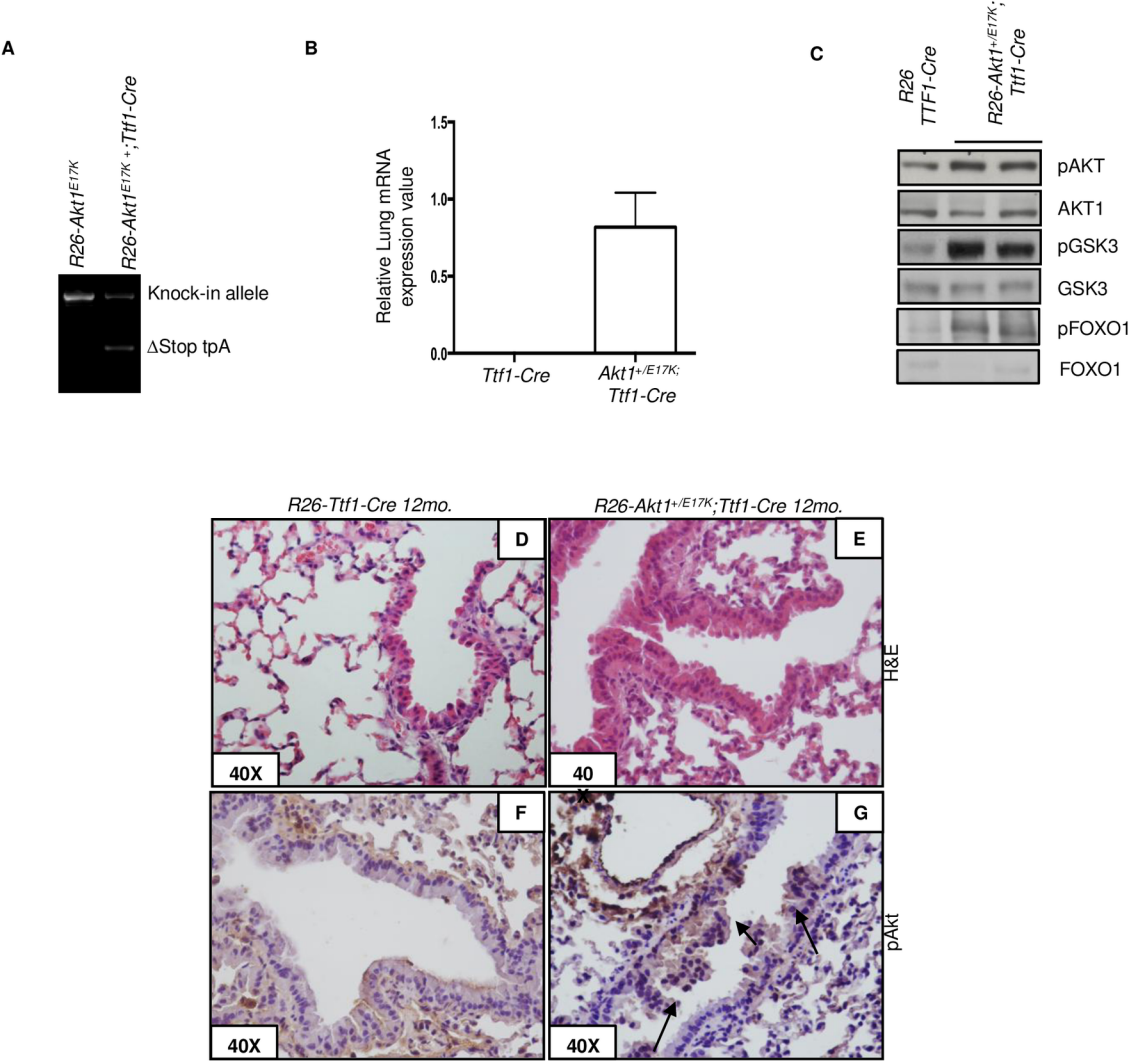
Phenotypic analysis of *R26-AKT1*^*E17K*^*; Ttf1-Cre* mice. **A**. PCR analysis of lung DNA from *R26-AKT1*^*E17K*^*; Ttf1-Cre* and control littermates. ∆Stop tpA: lox-P flanked transcription termination stop signal. **B.** Relative mRNA expression of human AKT1 by Q-RT-PCR on RNA of lungs from *R26; Ttf1-Cre*, *R26-AKT1*^*E17K*^*; Ttf1-Cre*. Data are presented from replicate analysis as the mean±SD. **C.** Representative immunoblot analysis of pAKT, total AKT1 and downstream signalling proteins in total protein extracts from whole lungs of *R26; Ttf1-Cre* and *R26-AKT1*^*E17K*^*; Ttf1-Cre* mice, respectively. **D-E.** Representative H&E staining of lungs from *R26;Ttf1-Cre* and *R26-AKT1*^*E17K*^*; Ttf1-Cre*, respectively. Magnification as indicated. **F-G.** Representative pAKT staining of lungs from *R26;Nkx2*.*1-Cre* and *R26-AKT1*^*E17K*^*;Nkx2*.*1-Cre*, respectively. Magnification as indicated.

In the first set of experiments, we crossed the *R26-AKT1*^*E17K*^ mice with *Ttf1-Cre*. Fig 2A shows the deletion of the loxP cassette in lung tissues of *R26-AKT*^*E17K*^*; Ttf1-Cre* mice; Fig 2B shows real-time RT-PCR expression of mutant AKT1 in the lung and Fig 2C shows increased levels of phosphorylated AKT and/or AKT substrates (GSK3α/β, FOXO1) in lungs from *R26-AKT*^*E17K*^*; Ttf1-Cre* mice compared to *R26-Ttf1-Cre* littermates. We measured AKT1 expression by RT-PCR in five additional tissues (tail, muscle, kidney, spleen, heart and liver) derived from R26-AKTE17K; Ttf1-Cre and R26-Ttf1-Cre littermate mice and the results are shown in S1 Fig. As shown, no expression is observed in tissues different from lungs.

Mice of 2 (n = 10), 6 (n = 8) and 12 (n = 11) months of age were sacrificed as end-points. *Ttf1-Cre* mice showed no evidence of disease up to 12 months of age (n = 7). Conversely, histo-pathological analysis revealed the presence of hyperplastic lesions in the lung of all 12 month-old *R26-AKT1*^*E17K*^*;Ttf1-Cre* mice (Fig 2D and 2E). These mice showed moderate hyperplasia of the bronchial and/or terminal bronchial epithelium with polarized nuclei. Alveolar epithelium was normal though atelectasis was observed in some mice. Immunostaining with anti-pS473 demonstrated increased levels of AKT activation in the hyperplastic lungs of *R26-AKT1*^*E17K*^*;Ttf1-Cre* mice compared with control or *Ttf1-Cre* mice (Fig 2F and 2G).

**Fig 3:**
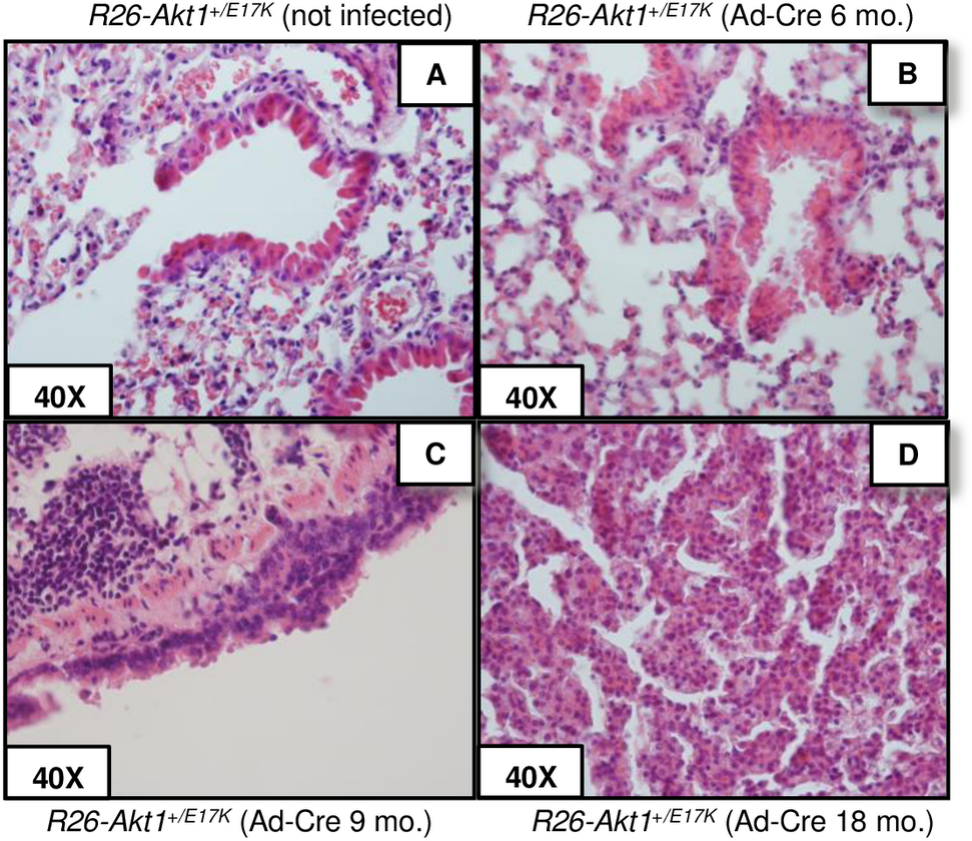
Phenotypic analysis of *R26-AKT1*^*E17K*^ mice infected with Ad-Cre. **A-D.** Representative H&E staining of lung epithelium of *R26-AKT1*^*E17K*^ mice treated with solvent alone (not infected) or infected with Ad-Cre (9 and 18 month-old, respectively). Magnification as indicated.

We also activated the mutant AKT1^E17K^ transgene by intranasal administration of Ad-Cre (10^6^, 10^7^ pfu) in 6–12 weeks-old *R26-AKT1*^*E17K*^ mice. After 6, 9 and 18 months (n = 8, 8 and 7, respectively) mice were sacrificed and lungs were analysed. We did not observe lung abnormalities in *R26-AKT1*^*E17K*^ mice treated with solvent alone (PBS) (n = 7) (Fig 3A). Conversely, virtually all *R26-AKT1*^*E17K*^ mice developed moderate bronchial and/or terminal bronchiolar hyperplasia at 9 months after Ad-Cre administration (Fig 3B and 3C).

To further characterise the lesions detected in transgenic *R26-AKT1*^*E17K*^ mice, we evaluated lung hyperplasia by analysing the number of epithelial layers in the bronchial epithelium and the percentage of hyperplasia on the whole area of 3 serial sections derived from representative *R26-AKT1*^*E17K*^ mice after 6 (n = 3) and 9 (n = 3) months from Ad-Cre administration (mice code #7a-#12a). As controls we made use of the same number of *R26-AKT1*^*E17K*^ mice treated with solvent alone (mouse code #1a-#6a), respectively. The quantification of epithelial layers and of hyperplastic areas was performed as described in Materials and Methods. Results are summarised in Table 1. Representative images of the scores assigned for the number of layers are shown in S2 Fig.

**Table 1:** Evaluation of hyperplastic lesions in *R26-AKT1*^*E17K*^ *mice*. Hyperplastic lesions were classified on the basis of the number of epithelial layers and the percentage of hyperplasia. Evaluation of epithelial layers, score 1: 1–2 layers of epithelial cells, score 2: 2–3 layers of epithelial cells, score 3: >4 layers of epithelial cells (n = 6 mice/group; 3 serial sections/mouse). Evaluation of the extent of hyperplasia, score 1: hyperplasia in 1–20% of total sections analysed; score 2: hyperplasia in 21–60% of total sections analysed; score 3: hyperplasia in 61–100% of total sections analysed (n = 6 mice/group; 3 serial sections/mouse). Lesions were classified according to a Combined score resulting from the sum of the score of layers and score of the percentage of hyperplasia observed (p<0.05; t-Student).

Mouse code	Treatment	Number of Epithelial Layers (score)	Percentage of hyperplasia (score)	Combined score	Mean of Final scores
#1a	Vehicle	1	1	2	2.5±0.3
#2a	Vehicle	2	1	3	
#3a	Vehicle	1	1	2	
#4a	Vehicle	1	1	2	
#5a	Vehicle	2	2	4	
#6a	Vehicle	1	1	2	
#7a	Ad-CRE	2	3	5	3.8±0.4
#8a	Ad-CRE	1	1	2	
#9a	Ad-CRE	2	2	4	
#10a	Ad-CRE	2	2	4	
#11a	Ad-CRE	2	1	3	
#12a	Ad-CRE	2	3	5	

Among the control mice Layer score was 1 for 4 out of 6 mice and 2 for 2 out of 6 mice. Global hyperplasia score was 1 for 5/6 and 2 for 1/6 mice. Conversely, of the *R26-AKT1*^*E17K*^ mice Layer score was 1 for 1/6 mice and 2 for 5/6 mice and Global hyperplasia score was 1 for 2/6, 2 for 2/6 and 3 for 2/6 mice. Finally, we found that *R26-AKT1*^*E17K*^ mice presented a Combined score of hyperplasia of 3.8±0.4 that was significantly higher than that in control mice (2.5±0.3) (n = 6/group; p<0.05).

Subsequently, we investigated whether the hyperplasic lesions observed were proliferative or senescence-related by immonostaining of Ki67 and p21. Results are summarised in Table 2 and representative images of staining for Ki67 and p21 in mouse lungs are shown in S3 Fig. We found that the average number of Ki67-positive nuclei in transgenic R26-AKTE17K mice (11.1±1.2, n = 5) was more than 2-fold increased compared to control mice (4.6±0.49, n = 3). Conversely, no significant difference of p21 staining was observed. These results suggest that the hyperplastic lesions induced by AKT1^E17K^ in the lung of transgenic mice are a result of increased cell proliferation.

**Table 2:** Evaluation of Ki67 and p21 staining in *R26-AKT1*^*E17K*^ *mice*

Mouse code	Treatment	Ki67 positive nuclei	p21 positive nuclei	Mean of Ki67 positive nuclei	Mean of p21 positive nuclei
#2a	Vehicle	2.3±1.8	0.7±1.7	4.6±0.49	2.4±0.4
#4a	Vehicle	6.2±2.4	1.5±1.3		
#5a	Vehicle	5.5±2.1	5.2±1.8		
#7a	Ad-CRE	21.4±10	2±1.5	11.1±1.2	2.8±0.5
#8a	Ad-CRE	6.1±2.4	6.6±5.8		
#10a	Ad-CRE	6.2±4.3	2±1.0		
#11a	Ad-CRE	16.4±6.4	2.4±2.2		
#12a	Ad-CRE	7.5±1.9	1±1.2		

Evaluation of Ki67 and p21 stainings was performed by counting positive nuclei in 10 fields at X400 magnification. (p<0.001; t-Student).

**Fig 4:**
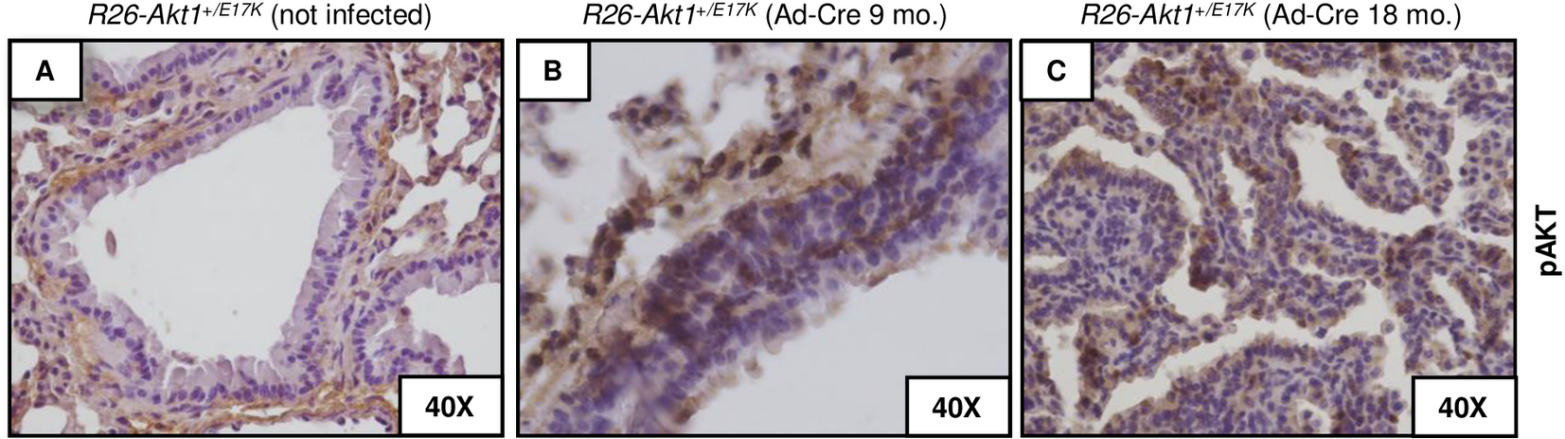
Immunostaining analysis of *R26-AKT1*^*E17K*^ mice. pAKT immunostaining of lung tissues from *R26-AKT1*^*E17K*^ mice treated with solvent alone (A) or infected with Ad-Cre after 9 and 18 months from infection, respectively (B, C).

It is of note that after 18 months from the treatment, two out of seven *R26-AKT1*^*+/E17K*^ mice treated with Ad-Cre developed a single nodule each, which was diagnosed as bronchio-alveolar adenocarcinoma with papillary pattern consisting of cords and nests of epithelial cells surrounded by sparse fibrovascular stroma (Fig 3D). Immunostaining analysis demonstrated high levels of pAKT in the hyperplastic and neoplastic lesions of *R26-AKT1*^*E17K*^ mice infected with Ad-Cre compared with lungs from non-infected mice (Fig 4A–4C).

In conclusion, it appears that the mutant AKT1^E17K^ allele induces moderate hyperplasia of bronchi and/or terminal bronchioli that, in the long-term and at low frequency, can progress to overt carcinoma.

### Mutant AKT1^E17K^ cooperates with chemical carcinogens

**Fig 5:**
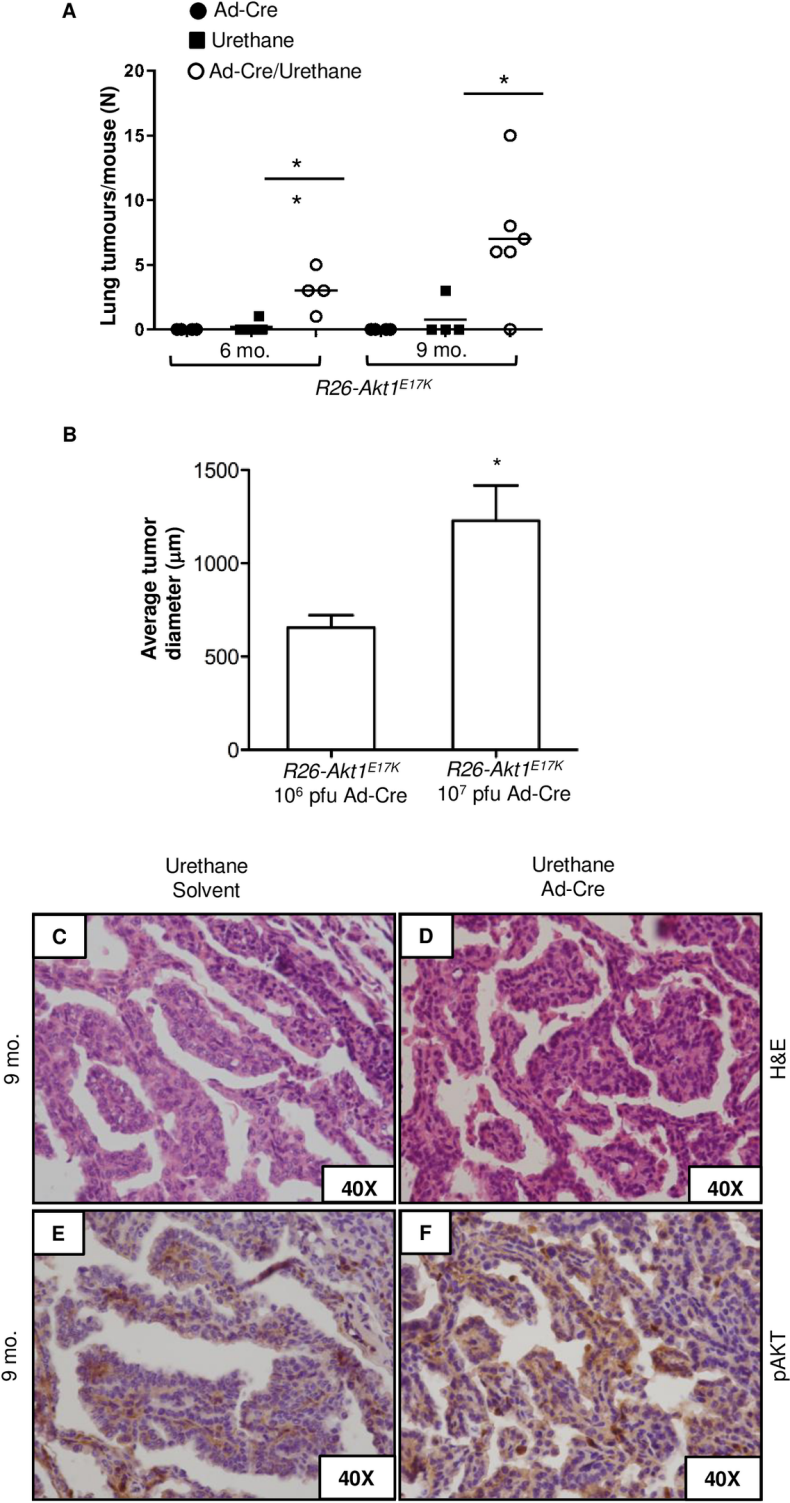
Mutant AKT1^E17K^ accelerates tumor formation induced by chemical carcinogens. **A.** Lung tumor multiplicity in *R26-AKT1*^*E17K*^ infected with Ad-Cre (6 and 9 months after the treatment, respectively). Each point represents one mouse; bars represent means ± SD. **p<0.01, *p<0.05. **B.** Diameter of lung tumors generated by urethane in *R26-AKT1*^*E17K*^ mice treated with increasing doses of Ad-Cre. Bars represent mean diameter of tumor ±SD. *p<0.05. **C, D.** Representative H&E staining of lung lesions developed in *R26-AKT1*^*E17K*^ mice treated with solvent or Ad-Cre, as indicated, 9 months after urethane administration. **E, F.** Phosporylated AKT staining of lung lesions developed in *R26-AKT1*^*E17K*^ mice treated with solvent or Ad-Cre, as indicated, 9 months after urethane administration. Magnification as indicated.

In human cells, AKT1^E17K^ promotes proliferation and migration when expressed in bronchial cells immortalized by the Adeno 12/SV0 hybrid virus [30]. To investigate whether AKT1 mutations are able to cooperate with other oncogenic hits also *in vivo*, we made use of ethyl carbamate (urethane), a chemical compound present in smoke that has been extensively used to experimentally model multistage lung carcinogenesis [41]. *R26 or R26-AKT1*^*E17K*^ littermates were either infected with Ad-Cre (10^6^−10^7^ PFU) or treated by solvent alone before being exposed to urethane (1mg/g) and analysed after 6 or 9 months. Multiplicity and size of tumors/lung were found to be significantly higher in *R26-AKT1*^*E17K*^ mice infected with Ad-Cre. Being relatively resistant to urethane, *R26-AKT1*^*E17K*^ mice that had not been previously exposed to Ad-Cre developed nodules in 1 out of 6 mice at 6 months and in 1 out of 4 mice at 9 months after urethane administration, respectively (Fig 5A). Conversely, almost all *R26-AKT1*^*E17K*^ mice that had been infected with Ad-Cre prior of urethane administration, presented lung nodules both after 6 (n = 4) and 9 (n = 6) months of treatment. The average number of tumors developed by mutant mice treated with urethane and infected with Ad-Cre was 3/mouse at 6 months and 7/mouse at 9 months; conversely, mice treated only with urethane developed <0.15 tumor/mouse at 6 months and of 1.6 tumors/mouse at 9 months (Fig 5A). Notably, the size of the tumors developed by mutant mice upon infection with Ad-Cre was dependent on the dose of Ad-Cre administered (Fig 5B).

Tumors generated by urethane in the *AKT1*^*E17K*^ background were diagnosed as bronchio-alveolar adenomas/adenocarcinomas that presented more frequently papillary pattern (Fig 5C and 5D). A final observation was that, unlike tumors generated in control mice that demonstrated little or moderate staining for pAKT, those generated in *R26-AKT1*^*E17K*^ mice presented a marked increase in pAKT staining (Fig 5E and 5F). Finally, we analysed *Kras* status in urethane-treated mice. DNA was extracted from microdissected formalin-fixed paraffine-embedded tumors of selected urethane-treated mice (2 mice treated with urethane alone and 4 mice treated with urethane plus Ad-CRE). The region spanning codon 61 in exon 3 of *Kras* gene was amplified and subjected to Sanger DNA sequencing. Sequence analysis of tumors demonstrated the presence of an A>G transition in codon 61 leading to Q61 replacement with R. See S4 Fig for representative chromatograms. These results indicate that AKT1^E17K^ may cooperate with the oncogenic events induced by urethane in mouse lung (i.e. *Kras* gain of function mutations), thus accelerating progression to overt tumors.

In the mouse, Clara cells line bronchiolar epithelium and express Clara cell-associated protein 10 (CC10) whereas alveolar type II (AT2) cells reside in the alveoli and express surfactant protein C (SPC) [42–44].

Therefore, to characterize the cells that constitute the lung lesions induced by AKT1^E17K^ in mouse, we performed indirect immunofluorescence for SP-C and CC10 on FFPE lung sections derived from *R26-AKT1*^*E17K*^ mice treated with Ad-CRE (4 mice of 9 months), urethane (2 mice of 9 months) and in *R26-AKT1*^*E17K*^ treated with urethane followed by Ad-CRE administration (2 mice of 9 months).

**Fig 6:**
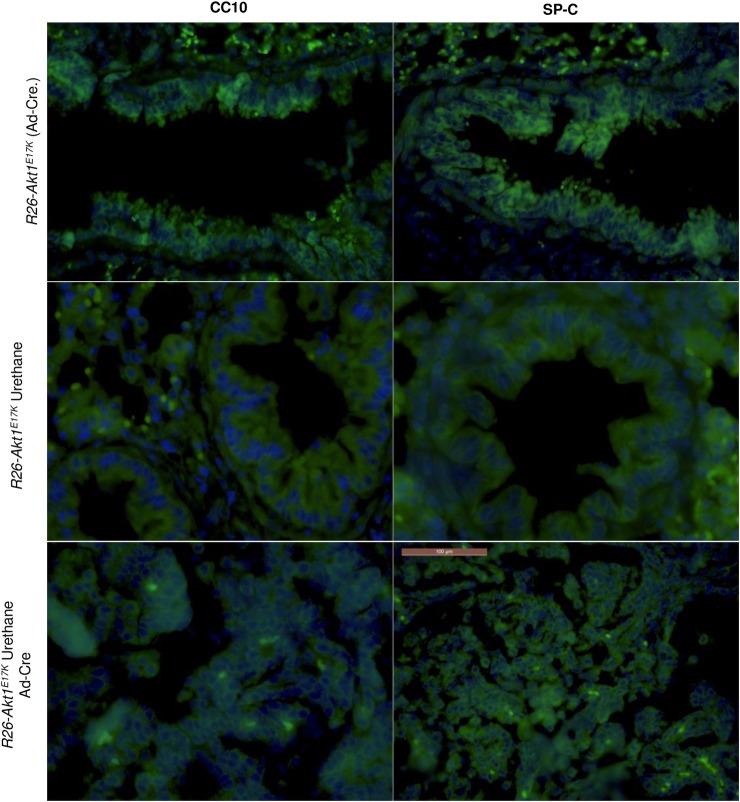
Indirect immunofluorescence for CC10 and SP-C in mouse lung. Representative immunofluorescence images of lung sections from *R26-AKT1*^*E17K*^ infected with Ad-Cre, treated with urethane or both. Panels A–C show lung sections analyzed using antibodies to CC10. Panels D–F show lung sections analyzed using antibodies to SP-C. Nuclei were stained with DAPI and are shown in blue. Magnification, 60X.

In agreement with previous studies [45,46], we found that lung tumors induced by urethane were strongly positive for SP-C but stained only weakly for CC10 (Fig 6), suggesting that urethane-induced tumors in these mouse strains show an AT2 phenotype.

Conversely, hyperplastic lesions initiated by *AKT1*^*E17K*^ in the lung were consistently positive for both SP-C and CC10. This immunophenotype is typical of i) a subset of variant Clara cells termed bronchioalveolar stem cells (BASCs) located at the bronchioalveolar duct junctions [47–49] and capable of differentiating into both Clara cells and AT2s [48] and ii) of a bronchiolar cell type (SPC^+^ bronchiolar epithelial cells, SBEC) that represents a SP-C+/CC10+ intermediate stage of Clara cell differentiation into AT2 cells [50].

## Discussion

The hotspot E17K mutation in the PH domain of AKT1 occurs in approximately 0.6–2% of human NSCLC patients. Recently, we have demonstrated that AKT1^E17K^ confers proliferative and pro-migratory characteristics to immortalized human bronchial epithelial cells. However, so far no murine model that recapitulates the E17K mutation identified in human lung cancer has been generated.

In this manuscript, we have genetically engineered a transgenic Cre-inducible murine strain in the *Rosa26* locus (*R26-AKT1*^*E17K*^) that allows targeted expression of mutant AKT1^E17K^ and studied its effects after activation in the lung. The most prominent findings are that in mice, AKT1^E17K^ promotes hyperplasia in bronchi and bronchi-alveoli and strongly cooperates with chemical carcinogens to induce malignancy. We found that, coincident with increased AKT signalling in lung epithelial cells, expression of mutant AKT1^E17K^ in the lung epithelium induced at high frequency moderate or severe hyperplasia of bronchi and/or terminal bronchioli that, in the long term, may progress to overt carcinoma in a very limited number of animals. It is of note that, hyperplastic lesions initiated by *AKT1*^*E17K*^ in the lung were positive for both Clara cell (CC10+) and AT2 (SP-C+) markers, an immunophenotype shared with rare BASCs at the bronchioalveolar duct junctions [47–49] and bronchiolar epithelial SBECs that exhibit Clara cell morphology and express both CC10 and SP-C [50].

Although AKT1^E17K^ mutation has, to our knowledge, not been reported in premalignant lung lesions isolated from patients, AKT signalling is frequently activated in bronchial pre-malignancy as shown by immunohistochemical studies of AKT phosphorylation in bronchial dysplasia [51]. Thus, the present study indicates the relevance of AKT1 activation as an early event that stimulates proliferation in the mouse lung, as observed in human cellular models [30].

Hyperplasia induced by mutant AKT1 in the mouse lung occurs later (on average, 12 months) compared with those induced by the oncogenic version of other genes within the PI3K pathway such as the upstream regulators EGFR (3–4 weeks) or KRAS^G12D^ (3 weeks). In addition to this, in *R26-AKT1*^*E17K*^ mice lung tumors appears with longer latency and lower incidence compared with mice expressing mutant *Egfr* (3–4 weeks from the activation of the oncogene) [52,53], *Pik3ca*^*H1047R*^ (12–60 weeks) [54] or *Kras*^*G12D*^ (in which the hyperplastic lesions progress to adenoma by 6 weeks, and less frequently to adenocarcinoma after 15 weeks of age) [38,55,56]. Conversely, analysis of *Pten* knock-out mice showed contradictory results [57,58] possibly depending on the specific promoter (CC10 and SP-C, respectively) used to delete the transgene or on the genetic background of the mouse strain [59]. In fact, *Pten* deficiency is not sufficient to induce histological abnormalities of the lung or to initiate lung tumorigenesis up to 12 months when induced by CCSP-driven Cre [57] but is able to induce macroscopic lung tumors after 13–20 months when induced by SP-C-driven Cre [58].

It is to be noted that the *R26-AKT1*^*E17K*^ mouse described here only partly recapitulates the human disease. On one hand, transgenic mice develop hyperplasia that in the long term (and infrequently) progresses to full-blown neoplastic lesions. This suggests that AKT1^E17K^ mutation, which in the human seems to be a weak driver mutation considering its rarity and absence in cell lines, represents a weak oncogene for the lung epithelium also in the mouse. However, at difference with what occurs in human patients, where the mutant allele AKT1^E17K^ is detected predominantly in SCC, the few tumors that develop in transgenic mice are ADC. This situation is reminiscent of mutant *Pik3ca* and/or *Pten* silencing both of which lead to ADC development in mice ^41, 44, 45^ but are preferentially detected in SCC in human.

Possibly, the observed difference in tumor histotype between human and mouse is dependent on the type of cell in which the oncogene is expressed. In fact, most oncogenes whose expression has been induced by Ad-CRE in mouse lung (*Kras*, *Egfr*, *Pik3ca*, *Pten* etc.) promote development of ADC, raising the possibility that Ad-CRE allows expression of transgenes only in ADC precursor cells. On the other hand, we have recently reported that human immortalized bronchial cells (BEAS-2B) transduced with a lentiviral vector carrying the activated AKT1^E17K^ mutant become tumorigenic and induce the appearance of poorly differentiated tumors [30].

A further consideration derives from the conclusions of chemical carcinogenesis experiments performed in *R26-AKT1*^*E17K*^ mice. In these experiments, AKT1^E17K^ strongly cooperated with urethane, a chemical carcinogen that induces A-G transitions at the codon 61 of the *Kras* gene [60–62]. The observed cooperation between oncogenically activated AKT1 and urethane is consistent with the evidence that AKT1 is the primary AKT isoform activated by mutant *Kras* in urethane-driven lung tumors in mice [62–64]. In addition it is of note that a synergy between activation of PI3K pathway and mutant *Kras* occurs in mice transgenic for mutant *Pik3ca* [54,65] or knock-out for *Pten* [11,57], indicating that signalling through both pathways is important for full oncogenic action in lung epithelial cells.

In summary, the conclusion drawn by the experiments reported here is that mutant AKT1^E17K^ is an oncogene that can initiate cancer in the mouse lung, though its oncogenic potency is apparently weaker than that of other oncogenes acting in the lung such as mutant *EGFR*, *Kras* or *PIK3CA*. Instead, the effects exerted by AKT1^E17K^ are more similar to those elicited by *PTEN* deficiency. This may account for the finding that AKT1 mutations are detected in a small fraction of lung cancer patients (0.5–2%) and are not selected for in human NSCLC cell lines.

## Supporting Information

S1 ARRIVE Checklist Guidelines Checklist. (PDF)

S1 Fig Relative mRNA expression of human AKT1 by Q-RT-PCR. (TIFF)

S2 Fig Score of the layers. (TIFF)

S3 Fig Immunostaining analysis of Ki67 and p21 in *R26-AKT1*^*E17K*^ mice. (TIFF)

S4 Fig Mutation analysis of KRAS in *R26-AKT1*^*E17K*^ *mice*. (TIFF)
