# Supplementary figures and images for: AKT1E17K Is Oncogenic in Mouse Lung and Cooperates with Chemical Carcinogens in Inducing Lung Cancer
Source: PLoS One. 2016 Feb 9;11(2):e0147334. doi: 10.1371/journal.pone.0147334 (PMC4747507; doi:10.1371/journal.pone.0147334)

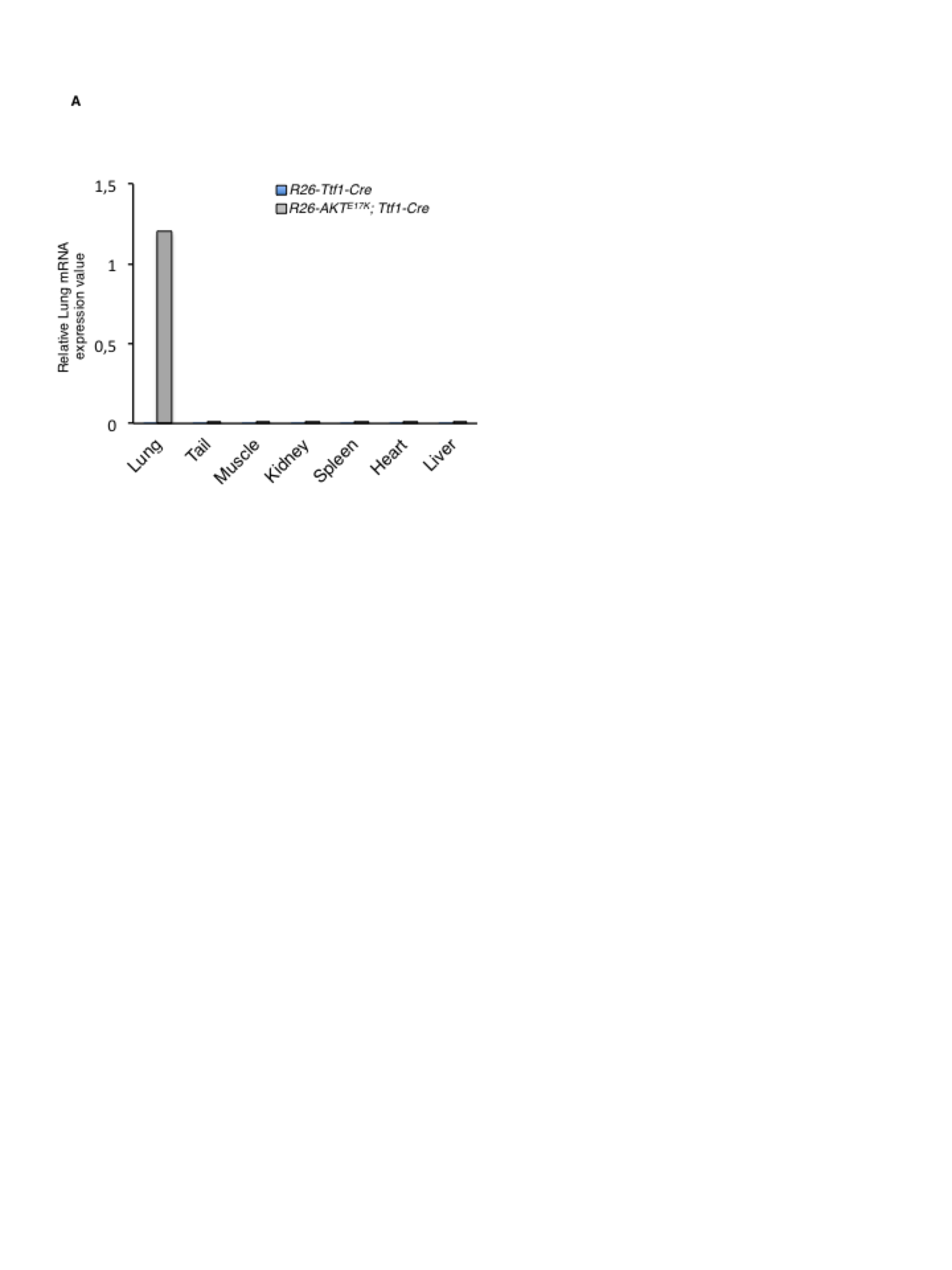

Supplement: S1 Fig — (TIFF) [file pone.0147334.s002.tiff]

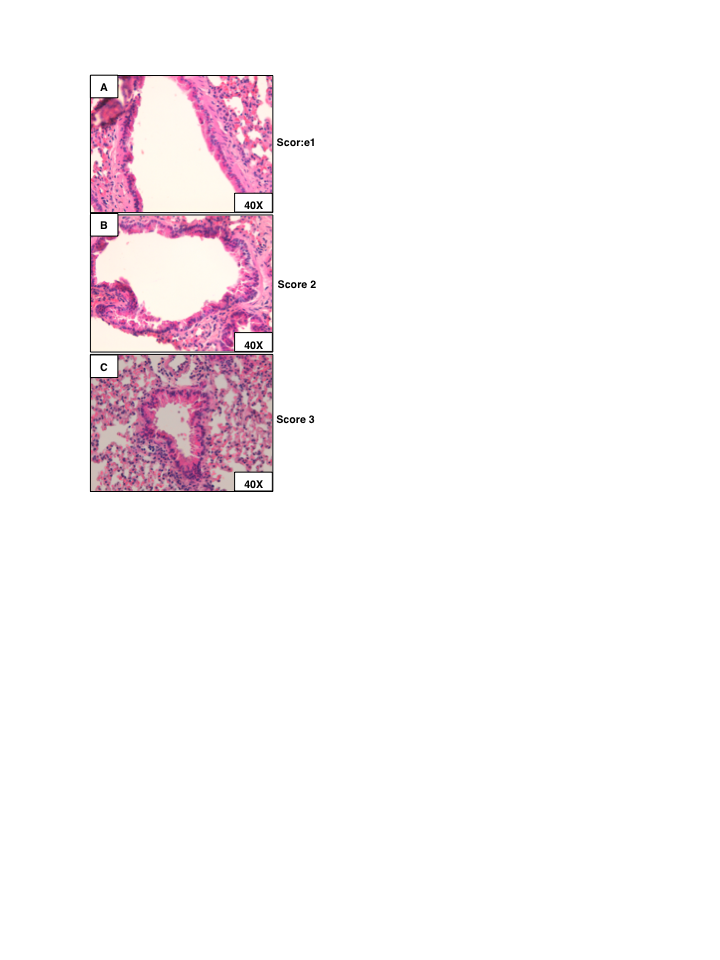

Supplement: S2 Fig — (TIFF) [file pone.0147334.s003.tiff]

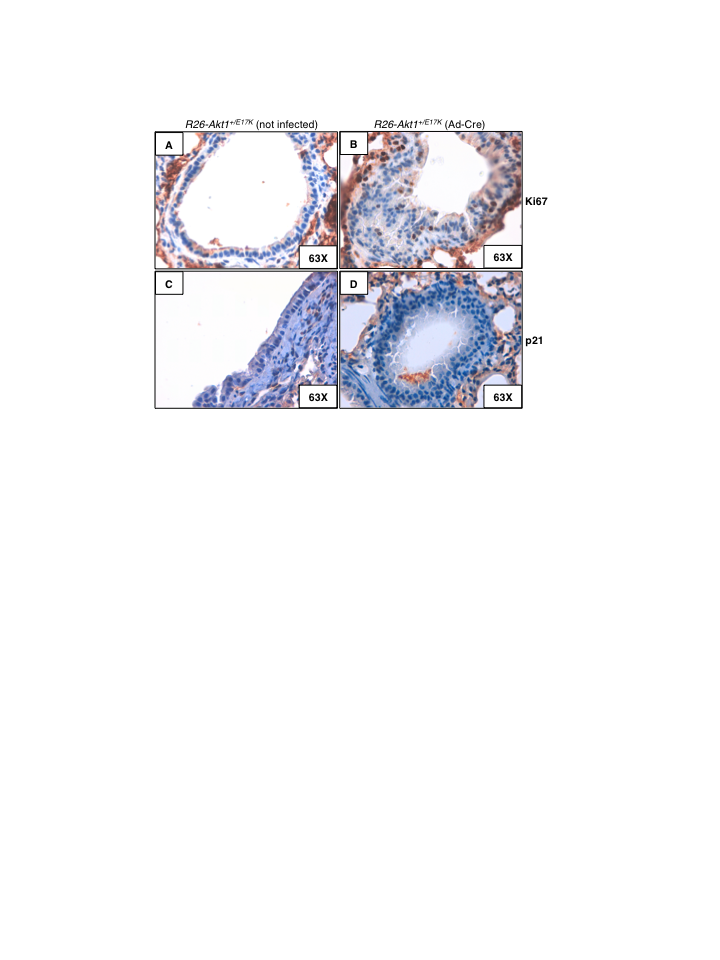

Supplement: S3 Fig — (TIFF) [file pone.0147334.s004.tiff]

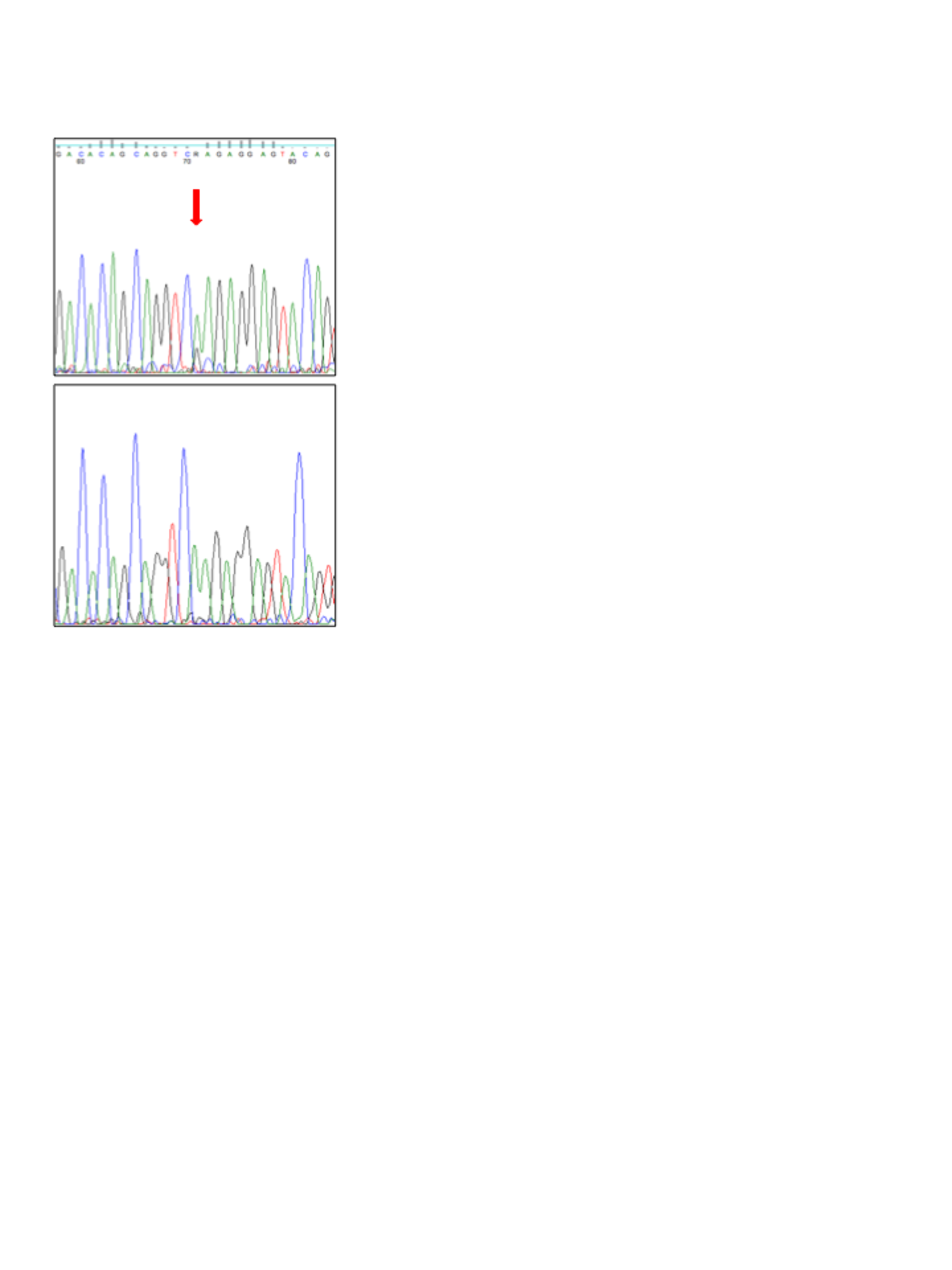

Supplement: S4 Fig — (TIFF) [file pone.0147334.s005.tiff]
